# A randomized controlled trial on the effects and acceptability of individual mindfulness techniques – meditation and yoga – on anxiety and depression in people with Parkinson’s disease: a study protocol

**DOI:** 10.1186/s12906-023-04049-x

**Published:** 2023-07-17

**Authors:** Jojo Yan Yan Kwok, Man Auyeung, Shirley Yin Yu Pang, Philip Wing Lok Ho, Doris Sau Fung Yu, Daniel Yee Tak Fong, Chia-chin Lin, Richard Walker, Samuel Yeung-shan Wong, Rainbow Tin Hung Ho

**Affiliations:** 1 School of Nursing, Li Ka Shing Faculty of Medicine, The University of Hong Kong, Pokfulam, Hong Kong SAR; 2 Department of Medicine, Pamela Youde Nethersole Eastern Hospital, Chai Wan, Hong Kong SAR; 3 Department of Medicine, Queen Mary Hospital, Pok Fu Lam, Hong Kong SAR; 4 Division of Neurology, Department of Medicine, School of Clinical Medicine, Li Ka Shing Faculty of Medicine, The University of Hong Kong, Pokfulam, Hong Kong SAR; 5 Department of Rehabilitation Sciences, The Hong Kong Polytechnic University, Hung Hom, Hong Kong SAR; 6 The State Key Laboratory of Marine Pollution, City University of Hong Kong, Kowloon Tong, Hong Kong SAR; 7 Northumbria Healthcare NHS Foundation Trust, North Tyneside General Hospital, Newcastle, UK; 8 Population Health Sciences Institute, Newcastle University, Newcastle, UK; 9 The Jockey Club School of Public Health and Primary Care, Faculty of Medicine, The Chinese University of Hong Kong, Shatin, Hong Kong SAR; 10 CUHK Thomas Jing Centre for Mindfulness Research and Training, The Chinese University of Hong Kong, Shatin, Hong Kong SAR; 11 Centre on Behavioral Health, The University of Hong Kong, Pokfulam, Hong Kong SAR; 12 Department of Social Work & Social Administration, The University of Hong Kong, Pokfulam, Hong Kong SAR

**Keywords:** Mindfulness, Psychological distress, Anxiety, Depression, Parkinson’s disease, Quality of life, Chronic illness care, Rehabilitation, Meditation, Yoga

## Abstract

**Background:**

Between 40 and 50% of patients with Parkinson’s disease (PD) experience anxiety and depression, associated with impaired physical function, high care dependency and mortality. Recently, the United States National Institutes of Health has urged the implementation of mindfulness practices in chronic illness care. Most research to date has examined the effects on chronically ill patients of complex interventions using a combination of mindfulness techniques. In PD patients, however, such complex modalities appear to hinder the technique mastery. Hence, the aim of this trial is to investigate the effects and underlying mechanism of individual mindfulness techniques among PD patients, as well as exploring participants’ experience in using individual mindfulness techniques as a lifestyle intervention for stress and symptom management.

**Methods:**

We will conduct an assessor-blind three-arm randomized waitlist-controlled trial with a descriptive qualitative evaluation. Up to 168 PD patients will be recruited from community settings and out-patient clinics, and randomized to meditation, yoga, or usual care group. Meditation and yoga sessions of 90-minute are held weekly for 8 weeks. Primary outcomes include anxiety and depression. Secondary outcomes include PD-related motor and non-motor symptoms and quality-of-life; and level of mindfulness and biomarkers of stress and inflammatory responses will be measured as mediating variables. All outcome evaluations will be assessed at baseline, 8 weeks, and 24 weeks. Following the intention-to-treat principle, generalized estimating equation models and path analysis will be used to identify the treatment effects and the mediating mechanisms. A subsample of 30 participants from each intervention group will be invited for qualitative interviews.

**Discussion:**

The study would also generate important insights to enhance the patients’ adaptation to debilitating disease. More specifically, symptom management and stress adaptation are highly prioritized healthcare agenda in managing PD. The research evidence will further inform the development of community-based, nurse-led compassionate care models for neurodegenerative conditions, which is complementary to existing health services.

**Trial registration:**

WHO Primary Registry – Chinese Clinical Trials Registry number: ChiCTR2100045939; registered on 2021/04/29 (https://www.chictr.org.cn/showproj.html?proj=125878).

## Background

Parkinson’s disease (PD) is the second most common neurodegenerative disease, affecting approximately 10 million people worldwide, and its estimated prevalence is expected to double by 2040 [1]. PD symptoms include four cardinal motor symptoms (resting tremor, rigidity, bradykinesia, and postural instability) and a range of non-motor symptoms including cognitive impairment, insomnia, constipation, and neuropsychiatric problems, which could severely limit patients’ daily function, independence, and HRQOL [2]. Adjusting to physical and psychosocial changes brought on by PD can be highly demanding and is associated with great psychological distress [3, 4]. Between 40 and 50% of PD patients experience anxiety and depression [5], and anxiety and anxious depression are the prominent psychopathological phenotype in PD [6]. Psycho-immunological studies of PD suggest that psychological distress and depressive symptoms are risk factors for psychiatric comorbidities and accelerated disease progression. Patients with high psychological distress reportedly experience greater disability, fast progression of physical symptoms, poor treatment compliance, increased comorbid conditions and mortality, high healthcare utilisation, and high caregiver distress [7].

While pharmacological intervention is used as the first-line treatment for more severe psychiatric manifestations in PD patients, its effects are suboptimal and even undesirable [8]. Mounting evidence suggests that psychological distress exerts a direct influence on PD pathogenesis by altering the hypothalamic-pituitary-adrenal (HPA) axis and potentiating inflammatory responses [9]. High cortisol levels provoked by HPA axis dysfunction and stress-induced upregulation of pro-inflammatory cytokines are associated with dopaminergic cell loss, increased symptom severity, and PD progression [10, 11]. These findings imply the potential benefits of psychological care not only for psychological outcomes and stress-related physiological parameters, but also for motor and non-motor symptoms of PD.

The United States National Institute of Health has urged the implementation of mindfulness practices in chronic illness care. Mindfulness, defined as ‘the awareness that emerges through paying attention on purpose, in the present moment, and nonjudgmentally to the unfolding of experience moment by moment’ [12], is a form of mental training that aims to improve an individual’s core psychological capacities, such as attentional and emotional self-regulation. Its positive effects on stress management have been demonstrated among populations with chronic conditions including psychiatric problems [13–15], cancer [16], chronic pain [17], and cardiovascular diseases [18]. A recent meta-analysis reported that mindfulness practices reduce physiological markers of stress, i.e., rebalancing the HPA axis with decreased cortisol levels, and improving inflammatory responses with reduced pro-inflammatory cytokines interleukin-6 (IL-6) and tumour necrosis factor-α (TNF-α) [19, 20]. Though conceptualisations of mindfulness vary, all highlight its role in enhancing attention and awareness of thought and emotion, and all contrast mindful information processing with automatic, habitual, or heuristic information processing [21]. The Liverpool Mindfulness Model elucidates the link between mindfulness and attention regulation processes on well-being [22]. The mindfulness training of attention skills underpins emotional and cognitive flexibility, bringing about the ability to maintain non-judgmental awareness of one’s own thoughts, feelings, and experiences which, in turn, will change the quality of one’s behaviour and lead to positive health outcomes and well-being [23].

With the popularity of mindfulness in wellness promotion, its research evidence on PD population remains relatively limited. Most mindfulness research examines the effects of the Mindfulness-Based Stress Reduction (MBSR) program conceptualised by Jon Kabat-Zinn. This group-based program focuses on progressive acquisition of mindfulness. An 8-week workshop is taught by certified trainers and entails weekly group meetings (2.5 h), a one-day retreat (7 h practice) between sessions 6 and 7, homework (45 min daily), and instruction in two core formal techniques: meditation and yoga postures [13–15]. Despite preliminary positive outcomes on emotional well-being having been reported in a few small-scale trials [24, 25], a 2017 systematic review concluded that the significant outcomes were contradicted and evidence to support MBSR practice for PD was inconclusive [26]. The inconclusive results may be related to the high dose and complex modalities (i.e., combined use of mindfulness techniques) of the MBSR program, aimed at enhancing participants’ extended immersive experience. A previous systematic review reported high dose (weekly ≥ 180 min) programs are associated with reduced adherence and increased physical intolerance caused by PD conditions [27], which may hinder PD patients’ skill mastery.

Indeed, mindfulness encompasses a family of practices, including meditation and yoga which are commonly used to promote well-being of patients with chronic illness [28]. Meditation is a form of relatively static mental training that starts with focused attention on an object to develop attentional stability and non-judgemental awareness of the current mental state [29]. This helps produce a deep state of relaxation, resulting in enhanced emotional and physical well-being. Yoga cultivates mindfulness experience through physical exertion [30]. The mental training starts with focused attention on various physical or emotional experiences arising from dynamic yoga movements. Apart from mental relaxation, the physical training of yoga may simultaneously target other major stressors in PD patients, including symptoms such as stiffness, muscle weakness and postural instability. However, the effects and acceptability of these individual mindfulness techniques remain underexplored in persons living with disabilities and chronic illnesses, such as PD.

To conclude, despite there being theoretical and empirical evidence suggesting the stress-relieving and health-enhancing effects of mindfulness interventions, a limited number of PD supportive interventions have focused on enhancing this attribute. Regarding the evolving stress-reducing effects of mindfulness, it is imperative to extend investigations into individual use of mindfulness practices, and to further examine their therapeutic value and underlying mediating mechanism, using both subjective and objective outcome measures, compared to usual care among persons with PD. This study aims to address these research gaps. The findings would provide a wider repertoire of evidence-based psychological interventions to support PD patients’ emotional health and symptom management.

### Aims and hypothesis

This study aims to:

1. examine the effects of individual mindfulness techniques – yoga and meditation – compared to usual care, on anxiety and depression, mindfulness, PD-related motor and non-motor symptoms, HRQOL and biomarkers of stress and inflammatory responses of PD patients over the 6-month study period;
2. investigate whether biomarkers and level of mindfulness function as mediating variables in the effects that the mindfulness practices have on anxiety and depression, respectively; and.
3. explore the experiences of PD patients in practising individual mindfulness techniques, and conditions that may influence their motivation, acceptability, and real-life practice.

The research hypotheses to be tested are:

1. PD patients who receive mindfulness interventions will report greater positive changes in anxiety and depression, mindfulness, PD-related motor and non-motor symptoms, HRQOL and biomarkers of stress and inflammatory responses on the completion of the intervention and at 4-month follow up, compared to the usual care group.
2. The effects of mindfulness practices on anxiety and depression are meditated by a reduced level of physiological markers of stress and improved level of mindfulness demonstrated at post-test assessment.

The exploratory research question is:

3. What are the participants’ experiences in using yoga and meditation as lifestyle intervention for psychological distress management, particularly the perceived effects, how and why mindfulness works or does not work, and conditions which may influence their motivation, acceptability, and real-life practice?

## Methods

### Study design and setting

**Fig. 1:**
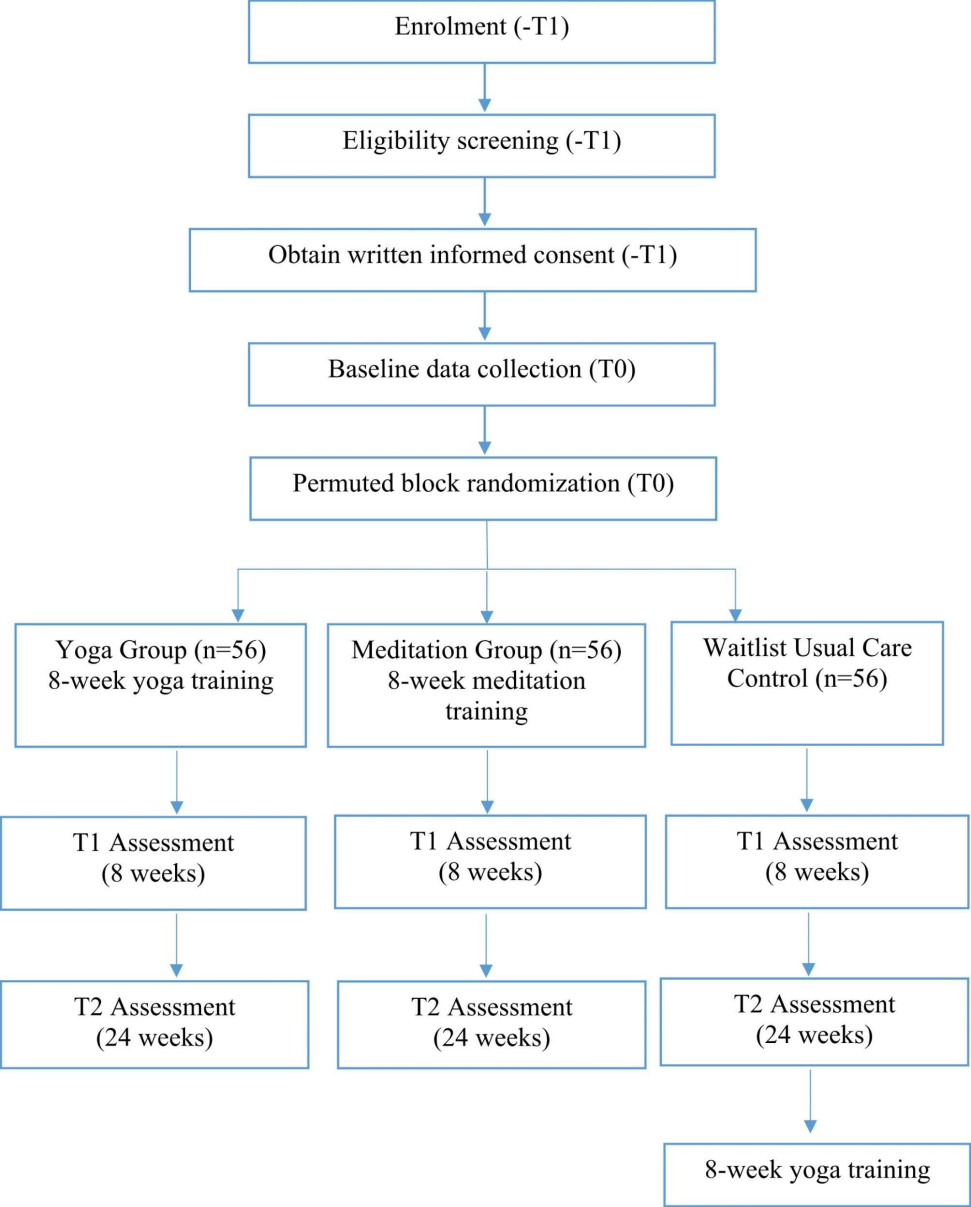
CONSORT flow diagram, indicating the schedule of enrolment, interventions and assessments of the study

This is a multi-centre, assessor-blinded, 3-arm randomised waitlist-controlled trial with sequential mixed-method design to evaluate the effect and acceptability of 8-week programs using individual mindfulness techniques (yoga alone and meditation alone), compared to wait-list usual care control. Figure 1 shows the CONSORT flow diagram. Repeated outcome measures will be assessed at three time points across the 6-month study period to quantitatively evaluate the immediate and sustained effects of each mindfulness technique on anxiety and depression and other physio-psycho-spiritual outcomes in PD patients, compared with the usual care control group. Assessment time points are: baseline (Prior to randomization, T_0_), 8 weeks (T_1_: 1-week post-intervention), and 24 weeks (T_2_: 4-month post-intervention).

Individual qualitative interviews with a purposive sample of 30 individuals from each intervention cohort will be conducted (1-month post-intervention) to explore their overall perception of using mindfulness for psychological distress management. The focus will be placed on how and why they perceive mindfulness practice to influence or not influence their psychological distress and acceptance of the intervention. The qualitative findings will be used to enhance the interpretation of the quantitative findings about the effect and mediating process of mindfulness on psychological distress and to inform the application of the findings [31]. The development-evaluation-implementation process of this study follows the Medical Research Council’s framework for complex interventions [32].

### Study participants

Eligible participants will be (i) diagnosed with idiopathic, mild-to-moderate PD as indicated by the Hoehn and Yahr Scale [33] Stages I to III (presented with unilateral to bilateral symptoms, with or without postural instability), (ii) aged ≥ 18 years, and (iii) able to give written consent. Individuals will be excluded if they: (i) have been regularly practicing instructor-led mindfulness interventions once a week or more during the past 6 months, (ii) are currently participating in any other behavioural or pharmacological trial, (iii) have significant cognitive impairment as indicated by the Abbreviated Mental Test lower than 6 [34], or (iv) have other contraindication or severe comorbidity that may limit their full participation (e.g. severe hearing or vision impairment, etc.).

### Sampling and recruitment strategies

Potential participants will be recruited from (i) neurological outpatient clinics at three regional hospitals: Queen Mary Hospital (QMH), Tung Wah Hospital (TWH), and Pamela Youde Nethersole Eastern Hospital (PYNEH), and (ii) PD support groups: Hong Kong Parkinson’s Disease Foundation (HKPDF), Hong Kong Parkinson’s Disease Association (HKPDA) and Community Rehabilitation Network (CRN). To enhance sample accessibility and representativeness, recruitment and intervention sites will cover three major local districts. Combinations of recruitment strategies will be used, including electronic mailing, printed flyers within the community centres and outpatient clinics, and dissemination of newsletters among patient support groups. Health talks will be held in community centres to aid recruitment, if necessary. All promotional materials and registration will be made accessible online.

### Allocation concealment, assignment of interventions and blinding

After baseline assessment, participants will be randomly assigned into one of the three groups on a 1:1:1 basis using the method of permuted block, with random block sizes between 3, 6, and 9, and will be informed of their assigned group by an independent person not involved in the assessment. The allocation list will be computer-generated by an independent researcher and concealed from other researchers and participants until the time of assignment.

### Study intervention

#### Meditation group

The mindfulness meditation intervention will be adapted from the structured eight-week mindfulness-based stress reduction (MBSR) program conceptualised by Jon Kabat-Zin [35] except the yoga component will be replaced by mindful movements with minimal physical exertion, and guided by our previous experiences of teaching mindfulness among local PD patients [36–38]. Previous PD literature reported that frequent and lengthy mind-body interventions (more than 3 times per week, with a total time > 180 min per week) were associated with reduced effectiveness owing to the decreased adherence and increased physical intolerance experienced by PD patients, and suggested a moderate dose of about 120 min per week as a feasible and practical mind-body practice for PD patients [27]. Hence, compared to the standard MBSR program (8 weekly 2.5-hour sessions, one all-day session between sessions 6 and 7, and daily 45-min homework), the tested meditation intervention will be modified to 8 weekly 90-min sessions, with 12 contact hours in total. Participants will be encouraged to perform 20-min home-based practice two times a week. The intervention will be delivered in neutral terminology free from any religious affiliation by a qualified MBSR instructor, and in a face-to-face group format of about 15–20 participants at local community centres.

The theme of each session reflects one of the 8 attitudes of mindfulness: non-judging, patience, beginner’s mind, trust, non-striving, acceptance, letting go, and generosity. The content and outline of the intervention are shown in Table 1, which has been adapted and modified from our previously tested meditation protocol for PD [38]. The weekly session consisted of standardized core elements of mindfulness practices: (i) guided meditation techniques (such as mindful breathing, body scan, etc.) focusing one’s attention on the breath, bodily sensations and non-judgmental awareness, and (ii) practicing being fully aware during everyday activities by using the breath as an anchor for the attention. If the instructor notices that participants start falling asleep (as indicated by head bobbing or snoring), various strategies will be used such as increasing voice volume, altering intonation, giving verbal cues, or changing meditation sequence, for example, chanting a simple mantra with participants. Paper handouts regarding the mindfulness techniques will be given to facilitate the learning and self-practice.

**T﻿able 1:** Theme and outline of the yoga and meditation programs

	Week 1		Week 2		Week 3		Week 4	
Program	Yoga	Meditation	Yoga	Meditation	Yoga	Meditation	Yoga	Meditation
Theme	Beginner’s mind: Introducing mindfulness		Patience: Allowing time for purifying mind, body and spirit		Non-judging: cultivating awareness towards inner and outer experiences		Acceptance: Embracing, exploring and expanding own limitations	
Outline	Warm up (Standing) 1. Mountain pose with balance shifting (centered, left, right, forward, heels) 2. Alternating between mountain and chair poses with synchronized breathing and arms movement Yoga sequence with mindful breathing 1. Crocodile pose 2. Sphinx pose 3. Cobra pose 4. Child pose Yoga nidra meditation	Guided body scan meditation in supine position Discussion: observation of daily stressful events	Warm up (as week 1) Yoga sequence (Standing) 1. Mountain pose 2. Side bend 3. Revolted forward bend 4. Lotus mudra 5. Cow face pose with forward bend 6. Lion’s breath Thoracic breathing Diaphragmatic breathing Mindful breathing Mindful eating	Guided body scan meditation in seated position Seated pose for meditation (On chair/ yoga block/ yoga mat) 1. Easy pose 2. Perfect pose 3. Half lotus 4. Full lotus 5. Diamond pose 6. Hero pose Mindful breathing Discussion: observation of daily pleasant events	Warm up (as week 1) Yoga sequence (Supine) 1. Mountain pose 2. Upward salute 3. Neck turning 4. Single leg raises with ankle rotation 5. Double leg raises 6. Bridge pose 7. Knees to chest pose 8. Corpse pose Santosha guided meditation in corpse pose Soham mantra meditation (seated)	Guided mindful breathing meditation in seated position Gentle mindful movement 1. Supine mountain pose 2. Supine upward salute 3. Knees to chest pose 4. Cat and cow pose 5. Balancing cat pose 6. Bridge pose 7. Supine spinal twist 8. Supine leg raises 9. Sleeping Vishnu pose 10. Front corpse pose 11. Half locust pose 12. Corpse pose Discussion: observation of daily unpleasant events	Warm up (as week 1) Yoga sequence (Standing) 1. Triangle pose 2. Warrior II pose 3. Side angle pose 4. Wide-legged forward bend Modified sun salutations 1. Mountain pose 2. Upward salute 3. Standing forward bend 4. Low lunge 5. Child pose 6. Tabletop pose 7. Knee, chest, chin pose 8. Cobra pose 9. Child pose 10. Downward-facing dog 11. Standing forward bend 12. Upward salute 13. Mountain pose Three-minute breathing space Yoga nidra meditation	Guided naming meditation in seated position Three-minute breathing space The stress-reaction cycle: respond vs react Discussion: observation of the responds towards unpleasant events

	Week 5		Week 6		Week 7		Week 8	
Program	Yoga	Meditation	Yoga	Meditation	Yoga	Meditation	Yoga	Meditation
Theme	Non-striving: Connecting to inner self without pushing		Generosity: Giving time, energy and attention to others while practicing self-compassion		Trust: Instilling confidence and loving-kindness		Letting go: Surrendering and being fully present	
Outline	Warm up (as week 1) Yoga sequence (Seated) 1. Diamond pose 2. Staff pose 3. Seated side bend 4. Cow face pose with forward bend 5. Lion’s breath 6. Camel pose 7. Rabbit pose Modified sun salutations (as week 4) Bee breath Cooling breath Om mantra meditation Yoga nidra meditation	Guided body meditation with mindful breathing in seated position Guided mindful walking Three-minute breathing space Mindful listening Discussion: observation of communication difficulties	Warm up (as week 1) Mindful walking Dyadic yoga sequence 1. Seated namaste (face-to-face) 2. Seated mountain pose with neck stretching (back-to-back) 3. Seated side bend (back-to-back) 4. Seated waist twisting (back-to-back) 5. Supported chair pose 6. Triangle pose (back-to-back) 7. Warrior II (back-to-back) 8. Tree pose (side-to-side) 9. Easy pose shoulder retraction partner Yoga nidra meditation in partner corpse pose	Guided open meditation in seated position Guided mindful walking Three-minute breathing space Mindful eating Mindful communication Discussion: cultivate awareness towards daily events and observation of body responds	Warm up (as week 1) Mindful walking Modified sun salutations (as week 4) Bee breath Alternating nostril breathing Om mantra meditation Loving kindness meditation in corpse pose	Guided loving-kindness meditation Mindful walking Three-minute breathing space Mindful living Discussion: cultivate loving-kindness towards self and the surrounding	Warm up (as week 1) Mindful walking Modified sun salutations (as week 4) Om mantra meditation Yoga nidra meditation	Open meditation in seated position Mindful breathing Mindful walking Three-minute breathing space Discussion: goal setting for continuous mindfulness practise in daily life

#### Yoga group

The mindfulness-based yoga intervention shares the same format and theme as the meditation intervention: 8 weekly 90-min sessions, with 12 contact hours in total. Participants will be encouraged to perform 20-min home-based practice two times a week. The intervention will be delivered in neutral terminology free from any religious affiliation by a qualified yoga and MBSR instructor, and in a face-to-face group format of about 15–20 participants at local community centres. The content and outline for each session are shown in Table 1, which has been adapted and modified based on our previously tested mindfulness yoga protocol for PD [37, 39]. The weekly session consisted of standardized core elements of yoga practices: (i) controlled breathing, (ii) mindfulness practice of yoga sequences (12 modified postures of sun salutations) with a focus on being aware of breath, bodily sensations and non-judgmental awareness during physical exertion, (iii) guided meditation techniques, and (iv) practicing being fully aware during everyday activities by using the breath as an anchor for the attention. Sharing of mindfulness practices experience will be facilitated by the instructor at the end of each session to consolidate learning and promote social interaction. Paper handouts regarding the mindfulness techniques will be given to facilitate the learning and self-practice.

#### Usual care group

The wait-list control group will continue with routine outpatient services (medication follow-up care with minimal health education on disease management/ drug care) and receive the tested yoga intervention [39] upon completion of the 6-month study period.

### Outcome measures

To detect the intervention effects, the following instruments will be used for outcome evaluation at baseline (Prior to randomization, T_0_), 1-week post-intervention (T_1_), and 4-month post-intervention (T_2_):

#### Primary outcomes

*Hospital Anxiety and Depression Scale (HADS)* (Chinese-Cantonese) will be used to measure anxiety and depression as primary outcomes [40]. HADS is a self-report questionnaire consisting of anxiety and depression subscales. Each subscale consists of seven items and each item is rated on a four-point scale. A high score represents a high level of psychological distress. The Cronbach alphas for the HADS-anxiety and HADS-depression subscales were 0.86 and 0.78 and construct validity is evident especially when no somatic symptom assessment is included, as such symptoms may be confused with Parkinsonian manifestations [41]. The test-retest reliability for subscales, as assessed by intraclass correlation coefficient (ICC), were 0.86 and 0.84 for the HADS-anxiety and HADS-depression [42].

#### Secondary outcomes

*Movement Disorder Society-Unified Parkinson’s Disease Rating Scale (MDS-UPDRS, Chinese)* will be used to measure PD-related motor and non-motor symptoms [43]. MDS-UPDRS consists of four parts, which examine: (i) non-motor experiences (cognitive impairment, hallucinations, depressed mood, anxious mood, apathy, and dopamine dysregulation), (ii) motor experiences of daily living, (iii) examination of motor symptoms (tremor, rigidity, bradykinesia, gait and postural instability), and (iv) motor complications (dyskinesia and motor fluctuations), respectively. Higher scores indicate greater disease severity. Cronbach’s alphas are 0.79–0.93 [44].

*Chinese Five-Facet Mindfulness Questionnaire (Short-form)* will be used to measure perceived mindfulness [45]. Using a 5-point Likert scale, this 20-item scale assess five mindfulness domains: observing, describing, acting with awareness, non-judgment of inner experience, and non-reaction to inner experience. Cronbach’s α is 0.82 [46].

*Parkinson’s Disease Questionnaire-8 (PDQ-8, Chinese)* will be used to measure PD-related HRQOL [47]. The 8-item scale yields a summary index score capturing disease-specific HRQOL regarding mobility, activities of daily living, emotional wellbeing, social support, communication, cognition, body discomfort and stigma. Higher scores indicate worse HRQOL. Cronbach’s α is 0.80 [48].

Plasma cortisol, IL-6 and TNF-α will be collected. To control for variations of cortisol levels over the circadian rhythm, all blood samples will be collected at 14:00 to 16:00, as cortisol level in this sub-period is relatively stable and highly correlated with mean 24-h cortisol level [49, 50].

The expectation on treatment credibility refers to the respondent’s expectation of the stress-reducing effect of the received intervention. A 4-item questionnaire will be used, rating: (i) reasonableness of treatment, (ii) opinion of the therapist, (iii) expectation for improvement, and (iv) likelihood to recommend the treatment to others, on a 10-point scale (higher scores indicating higher expectation) [51].

Socio-demographic and clinical data will be documented using a self-reported questionnaire, which covers participant profile (age, gender, marital status, educational level, religiosity, and financial status) and clinical data (onset time, PD staging, medication record, history of psychiatric disturbance, presence of comorbidity, history of rehabilitation service utilization). All these will be documented and considered as control variables in the data analysis.

### Data collection procedures

The researcher will contact the referred/registered potential participants by phone and screen for their eligibility. Eligible participants will be invited to a face-to-face interview at a community center. The research team will first obtain written informed consent from the participants, followed up by demographic and baseline assessments. Questionnaires in e-format will be administered using an iPad: a well-accepted data collection approach as shown by our previous PD trials [39]. A research assistant will help participants with difficulties in filling out the questionnaires. All assessments will be conducted during the ‘on’ state of medications to minimize motor fluctuations. Blood samples will be collected by qualified personnel. Neurological motor examinations will be conducted by qualified personnel with an MDS-UPDRS certificate.

Qualitative data will be collected to explore participants’ experiences. A purposive sampling of 30 participants with diversified socio-demographic characteristics from each of the intervention groups will be invited for individual interviews. This sample size complies with various guidelines which suggest that a sample of 25 participants is adequate to reach data saturation and the sample of a qualitative study should lie within 50 [52]. The pre- and post-test difference of HADS scores will be used as the pre-defined criterion, with ten participants in each range of the 0-35th, > 35-70th, and > 70th percentile. If saturation is not reached, one more case will be recruited from each percentile until no more new findings emerge. The interview guide (Table 2) will focus on exploring participants’ experience in using the mindfulness practice as a lifestyle intervention for stress management, particularly the perceived effects, how and why they work or do not work, and conditions that may influence their motivation, acceptability, and real-life practice.

**Table 2:** Interview guide for qualitative interview

Questions	Probes
1. How do you feel about the 8-week mindfulness training? 2. How has your mood changed while you were participating in the 8-week mindfulness training? 3. How is your current mood different from the situation before you participated in the 8-week mindfulness training? 4. You have mentioned that you have improved mood after participating in the 8-week mindfulness training. What are the possible reasons leading to such changes? OR You have mentioned that your mood remains the same after participating in the mindfulness training. Why is that? What are the possible reasons? 5. Will you recommend the 8-week mindfulness training to your friends with Parkinson’s disease or with mood disturbance? Why? 6. Will you consider doing regular mindfulness practice in the future? Why?	Acceptability and tolerance Mood changes - Anxiety - Depressive symptoms - Number and duration of psychological distress - Physical fitness - Motor symptoms (tremor, rigidity, bradykinesia, gait and postural instability) - Non-motor symptoms (cognition, hallucinations, depressed mood, anxious mood, apathy, dopamine dysregulation) - Perception on psychological distress - Perceived effectiveness of mindfulness training

### Sample size estimation

To account for multiplicity, we consider 2.5% as the nominal level of significance by Bonferroni adjustment. Taking psychological distress as the primary outcome, a previous systematic review reported an effect size of 0.4 for meditation compared to cognitive behavioral therapy control [53], while our previous clinical trial reported an effect size of 0.6 for yoga compared to exercise control [39]. Taking a conservative approach, although the comparison is made to usual care, we anticipate a moderate effect size of 0.6 for the primary outcome. Using power analysis software Gpower 3.1, assuming an attrition rate of 20%, 168 participants will give a three-arm (56 each arm) RCT 80% power to detect an effect size of 0.6 at 2.5% level of significance.

### Data analysis

Descriptive statistics will be used to summarize the socio-demographic characteristics and clinical data (as listed above) of all subjects at all time points. Generalized estimating equations (GEE) with unstructured working covariance matrix at the individual level with repeated measurements will be performed to compare all outcomes [1–5] between groups (i.e., time effect, group effect, group*time effect). An intention-to-treat principle will be adopted. Multiple imputations will be used to account for missing data, and reporting will follow the guideline published in *BMJ* [54]. Sensitivity analysis will be performed to compare the results between complete and non-complete cases. All demographic and clinical variables (e.g. age, gender, religiosity, expectation on treatment credibility, etc.) will be accounted for in the analysis. Both crude and adjusted GEE model analyses will be performed. All statistical tests will be two-tailed and statistical significance will be set at 0.025. IBM SPSS 25.0 will be used.

**Fig. 2:**
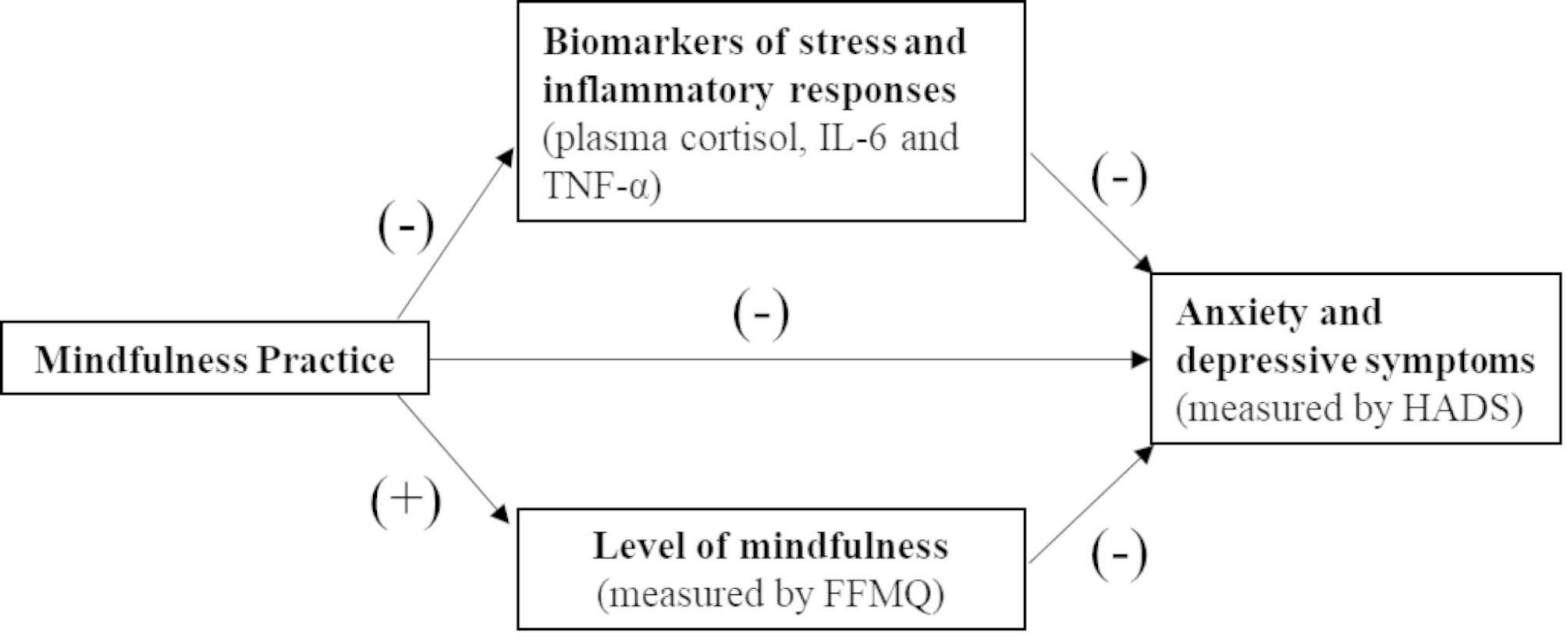
Mediating process of mindfulness on psychological distress

Path analysis will be used to examine the mediating role of biomarkers and level of mindfulness on the effect of mindfulness training on psychological distress. Based on the hypothesized mediation model (Fig. 2), a baseline path model will be first built, then a path model with the same structure for T1 will be added and linked by intra-correlations among the same variables. The mediation effects of biomarkers and level of mindfulness on the effect of mindfulness training on psychological distress will be assessed through the significance of the corresponding products of indirect path coefficients from mindfulness training to psychological distress, respectively [55]. In view of the possibility of violation of normality assumption, a bootstrapping approach, instead of the conventional multivariate delta method, will be used to estimate the standard errors and the confidence intervals of the product terms (mediation effects) [56]. The path analyses and the bootstrapping will be performed using IBM SPSS 25.0.

For qualitative data, the audio-taped interview will be transcribed verbatim, and managed by NVivo 12.0. Two experienced qualitative researchers will carefully and inductively review the transcribed content. Inductive thematic analysis will be used to code the data [57]. The coded units will be sorted into categories and subcategories and analyzed for recurrent themes and patterns. Within-case and across-case analyses in each group of participants with a similar change in HADS score will be conducted and followed by cross-group analysis. Audit trail will be conducted to maintain data credibility. The emerging categories will be reviewed for resonance with the quantitative findings. The qualitative findings will serve a complementary purpose in concluding the effects and acceptability of using individual mindfulness techniques for stress management among PD patients.

### Ethics and dissemination

This research has been approved by the Institutional Review Board of the University of Hong Kong/ Hospital Authority Hong Kong West Cluster (HKU/HA HKW IRB) (UW 19–446) and registered in the WHO Primary Registry – Chinese Clinical Trials Registry (ChiCTR) (ChiCTR2100045939). The study aims, intervention content, voluntary participation, right to withdraw at any time will be explained verbally and outlined in detail on an information sheet. The researcher will emphasize that their decision to join/refuse/withdraw from the study will not affect the routine care and services provided by the community centers/neurology outpatient clinics. Each participant will be given a subject code and no identifiable information will be presented in the data file to protect participants’ confidentiality. All the data collected will be stored in a secure place and can only be accessed by the research team members. The trial will be conducted in accordance with the Declaration of Helsinki, and reported in accordance with the CONSORT guideline and its extension to non-pharmacological interventions [58]. Results will be disseminated via presentations at scientific conferences, peer-reviewed publications, public engagement events, stakeholder organisations, patient support groups and other forms of media where appropriate. The investigators will be involved in reviewing drafts of the manuscripts, abstracts, press releases and any other publications arising from the study.

## Discussion

PD is a progressive, incurable, and irreversible neurodegenerative disease that affects both motor and psycho-cognitive function. Oxidative stress has been suggested to play a key role in PD progression [59]. Increasing attention has, therefore, been directed to reversible preclinical manifestations such as psychological distress management. Mounting evidence has consistently highlighted the high prevalence of psychological distress and identified it as a significant risk factor for accelerated disease progression; however, the current management efforts highly cluster on motor training, and psychological care is always a neglected area in the research arena. Hence, this study will examine the effects of mindfulness interventions on addressing psychological distress and other health-related outcomes in this clinical cohort.

Despite the public recognition and theoretical assumptions on the stress-reducing effects of mindfulness, research evidence on this topic is relatively less compelling, and there is a need for a more stringent scientific evaluation of this lifestyle intervention using subjective and objective stress outcomes. Examining such effects on physiological markers of stress (cortisol and pro-inflammatory cytokine levels) will shed light on the homeostatic effects of mindfulness on the dysregulating of the hypothalamic-pituitary–adrenal system and inflammatory responses. The investigation of underlying mediating mechanisms would advance the knowledge which explain the effects of mindfulness-based practice. Based on the Liverpool Mindfulness Model [22], instant attentional control mechanisms are the central core processes of mindfulness, which include the attentional awareness of one’s cognitive and emotional states and the resulting ability to respond to them in a flexible way. Attentional awareness and a non-judgmental mental stance are considered as two main contributors when developing a mindful approach to one’s life. Malinowski [22] and Shapiro, Carlson [60] suggested that without refining attentional skills and developing the ability to maintain a stable focus of attention for some time, profound growth and development of mindfulness skills would be challenging. Indeed, recent mindfulness literature also highlighted it is crucially important to consider what it takes for people to be interested in, and willing to practice, mindfulness. Although the practice of mindfulness could be conceptually simple – just focus on the sensations of your breathing and maintain a non-judgmental, open awareness of all arising mental events – the processes of developing these mindfulness skills could vary among individuals, in particular, people living with disabilities and chronic illnesses, such as PD. It is therefore important to explore what are the participants’ experiential experiences and acceptability towards individual mindfulness techniques. This study will examine two common individual mindfulness techniques – meditation and yoga, ‘Mindfulness in stillness’ and ‘mindfulness in motion’, respectively. Exploring the experience and acceptability of PD patients for using individual mindfulness techniques as lifestyle intervention to improve emotional distress and symptom management will also facilitate the subsequent knowledge transfer. This study aims at address all these research agendas. Such knowledge is compelling to advance the science on using complementary therapy in PD management.

The study would also generate important insights to enhance the patients’ adaptation to debilitating disease. More specifically, symptom management and stress adaptation are highly prioritized nursing agenda in managing PD. Understanding the stress-reducing effects and acceptability of different mindfulness practices is crucial to inform the treatment options for anxiety and depression of PD patients. The self-help nature of such practice also implies its high relevance to enrich the primary care for this clinical cohort. By focusing on the lifestyle intervention that is acceptable among PD patients, evidence derived from this study will be readily translatable to real-life practice through a territory-wide campaign for promoting mental health and PD rehabilitation. Results will support the implementation and extensions of evidence-based mindfulness practices in PD care worldwide. The research evidence being generated will also inform the further development of community-based and compassion-focused care models in promoting chronic disease adaptation, which is complementary to existing healthcare services.

### Limitations

There are several potential limitations of the study. First, even though a range of recruitment strategies will be used to approach PD patients in various settings, the recruited participants may be more active in community engagement. This a possible source of selection bias which may limit the generalizability of the study findings to the less active group. Second, blinding the participants is inapplicable owing to the nature of the behavioral interventions, which may induce risks of bias in terms of performance, attrition, and evaluation. To minimize bias stemming from expectation, study participants will be blinded to study hypotheses and details of the study manuals. The information provided in the informed consent form followed the principle of equipoise by declaring uncertainty about the superiority of the treatment effect of both intervention arms. All participants will be reminded not to disclose their group status to the study assessor at any time point. Third, the validity of the study may be threatened by a high attrition as the intervention requires the participants to attend 8 face-to-face intervention sessions plus 3 assessment interviews, particularly during the coronavirus pandemic. To secure their attendance and ensure safety, appropriate public health control measures will be enforced in line with government regulations, such as mandatory mask-wearing and social distancing. If there is an unexpected lockdown, the research team will call upon an ad hoc meeting to discuss possible solutions, including, but not limited to, change of venue, schedule, and using a hybrid on-site/virtual delivery mode [61]. To reduce attrition among the control group, participants will receive the tested yoga intervention upon completion of the three assessment interviews over the 6-month study period. Finally, participants who have attended and completed the assessment interviews will receive HKD 50/50/100 cash incentive at the baseline, T1 and T2 follow-ups, respectively.

## Conclusion

This study addresses the highly prevalent psychological distress among PD patients by adopting a psycho-behavioral approach using mindfulness techniques. With the growing evidence to suggest the individual use of either yoga or meditation in PD patients, the current study adopts a stringent research design to evaluate the health effects using both subjective and objective stress outcomes, mediating mechanisms, and patients’ experience of such practice. This comprehensive approach can contribute to current knowledge of the clinical values and acceptability of different forms of mindfulness practices as community-based and compassion-focused lifestyle interventions to help PD patients cope with the physical and psychosocial challenges as they live with the disease. Adopting a mixed-method design can further enhance the interpretation of the quantitative findings and inform future applications in the healthcare service.

## Data Availability

The datasets generated or analysed from the current study are not publicly available due to the privacy of individuals that participated in the study but are available from the corresponding author on reasonable request.
